# Improved retinal function in RCS rats after suppressing the over-activation of mGluR5

**DOI:** 10.1038/s41598-017-03702-z

**Published:** 2017-06-14

**Authors:** Jiaman Dai, Yan Fu, Yuxiao Zeng, Shiying Li, Zheng Qin Yin

**Affiliations:** 1 Bioengineering College, Chongqing University, Chongqing, 400040 China; 2 Southwest Hospital/Southwest Eye Hospital, Third Military Medical University, Chongqing, 400038 China; 3 Key Lab of Visual Damage and Regeneration & Restoration of Chongqing, Chongqing, 400038 China

## Abstract

Müller cells maintain retinal synaptic homeostasis by taking up glutamate from the synaptic cleft and transporting glutamine back to the neurons. To study the interaction between Müller cells and photoreceptors, we injected either DL-α-aminoadipate or L-methionine sulfoximine–both inhibitors of glutamine synthetase–subretinally in rats. Following injection, the a-wave of the electroretinogram (ERG) was attenuated, and metabotropic glutamate receptor 5 (mGluR5) was activated. Selective antagonism of mGluR5 by 2-methyl-6-(phenylethynyl)-pyridine increased the ERG a-wave amplitude and also increased rhodopsin expression. Conversely, activation of mGluR5 by the agonist, (R,S)-2-chloro-5-hydroxyphenylglycine, decreased both the a-wave amplitude and rhodopsin expression, but upregulated expression of G_q_ alpha subunit and phospholipase C βIII. Overexpression of mGluR5 reduced the inward-rectifying potassium ion channel (K_ir_) current and decreased the expression of K_ir_4.1 and aquaporin-4 (AQP4). Further experiments indicated that mGluR5 formed a macromolecular complex with these two membrane channels. Lastly, increased expression of mGluR5 was found in Royal College of Surgeons rats–a model of retinitis pigmentosa (RP). Inhibition of mGluR5 in this model restored the amplitude of ERG features, and reduced the expression of glial fibrillary acidic protein. These results suggest that mGluR5 may be worth considering as a potential therapeutic target in RP.

## Introduction

Photoreceptor degeneration is poorly understood, but can lead to blindness in retinitis pigmentosa (RP). Many previous studies have demonstrated that Müller cells–the principal radial glial cells across the retina–play an important role in both the healthy and diseased retina^1^. The role of Müller cells includes the uptake and recycling of glutamate released by photoreceptors^2^.

Extracellular glutamate acts on postsynaptic neurons via both ionotropic (iGluRs) and metabotropic glutamate receptors (mGluRs)^3, 4^. Eight mGluRs (mGluR1–8) have been cloned, and all of these subtypes (except mGluR3) have been identified in the retina^4^. mGluR1 and mGluR5 are known as ‘Group I’ mGluRs, and are G protein-coupled to phospholipase C (PLC) activation, leading to calcium ion (Ca^2+^) release from internal stores. mGluR1 and 5 have been extensively studied in the brain and spinal cord^5–7^ but much less so in the retina, where the expression of mGluR5 is localized to bipolar cells and amacrine cells^8, 9^. In cultured Müller cells, mGluR5 receptors are known to modulate the transcription and translation of an inward-rectifying potassium ion channel, K_ir_4.1^10^. Furthermore, mGluR5 is activated in a rat model of chronic ocular hypertension (COH), leading to suppression of K_ir_ currents and reduced expression of K_ir_4.1^11^.

The proper function of the glutamatergic synapse is dependent not only on presynaptic release machinery and postsynaptic receptors, but also on the effective removal of glutamate from the synaptic cleft (also preventing neuronal excitotoxicity). The bulk of glutamate is removed from the cleft by presynaptic excitatory amino acid transporter 5 (EAAT5) on photoreceptors^12^. However, Müller cells also play a vital role in the recycling of the neurotransmitter, via the ‘glutamate-glutamine cycle’. Glutamate is taken up by Müller cells via excitatory amino acid transporter 1 (EAAT1, also known as glutamate/aspartate transporter [GLAST]) and is then converted into glutamine by glutamine synthetase (GS), which is a glia-specific enzyme^1, 13^, and transported back to the neurons for recycling into glutamate.

Despite its minority role at the synapse, the inhibition of EAAT1 still affects the retinal response to light, causing a reduction in the amplitude of the scotopic electroretinogram (ERG) b-wave^14–16^, which suggests an effect on the activity of rod bipolar cells^17–19^. In addition, inhibition of GS by intravitreal injection of a GS inhibitor leads to decreased amplitude of the b-wave, but not the a-wave^20^, which reflects the light-induced responses of photoreceptors^21^. However, induction of glial dysfunction via the inhibition of GS affects the assembly and mosaic arrangement of photoreceptors^22, 23^. Most importantly, the selective ablation of Müller cells results not only in apoptosis of the photoreceptors, but also in decreased amplitudes of both a- and b-waves in the ERG^24, 25^.

Here, we studied whether the light response of photoreceptors is affected by the inhibition of GS in Müller cells. We found that the ERG a-wave was attenuated after subretinal injection of GS inhibitor. We then investigated the cause of this a-wave attenuation. Glutamate-mediated excitotoxicity is known to be linked to photoreceptor loss in retinal degeneration^26^. And over-activation of mGluRs has been reported to contribute to the pathogenesis of glaucoma and other neurological disorders^11, 27^. We therefore hypothesized that mGluR5 over-activation, due to excessive synaptic glutamate, might mediate the effects of GS inhibition. Our results showed that mGluR5 was activated after GS inhibition, and a-wave amplitude was regulated by mGluR5 activity. In addition, mGluR5 was over-activated during the degeneration of RCS rats (a model of RP). Moreover, inhibition of the over-activated mGluR5 in this model restored the amplitude of the ERG. These results suggest that mGluR5 may be worth considering as a potential therapeutic target in RP.

## Results

### Activation of mGluR5 reduced the amplitude of ERG features

**Figure 1:**
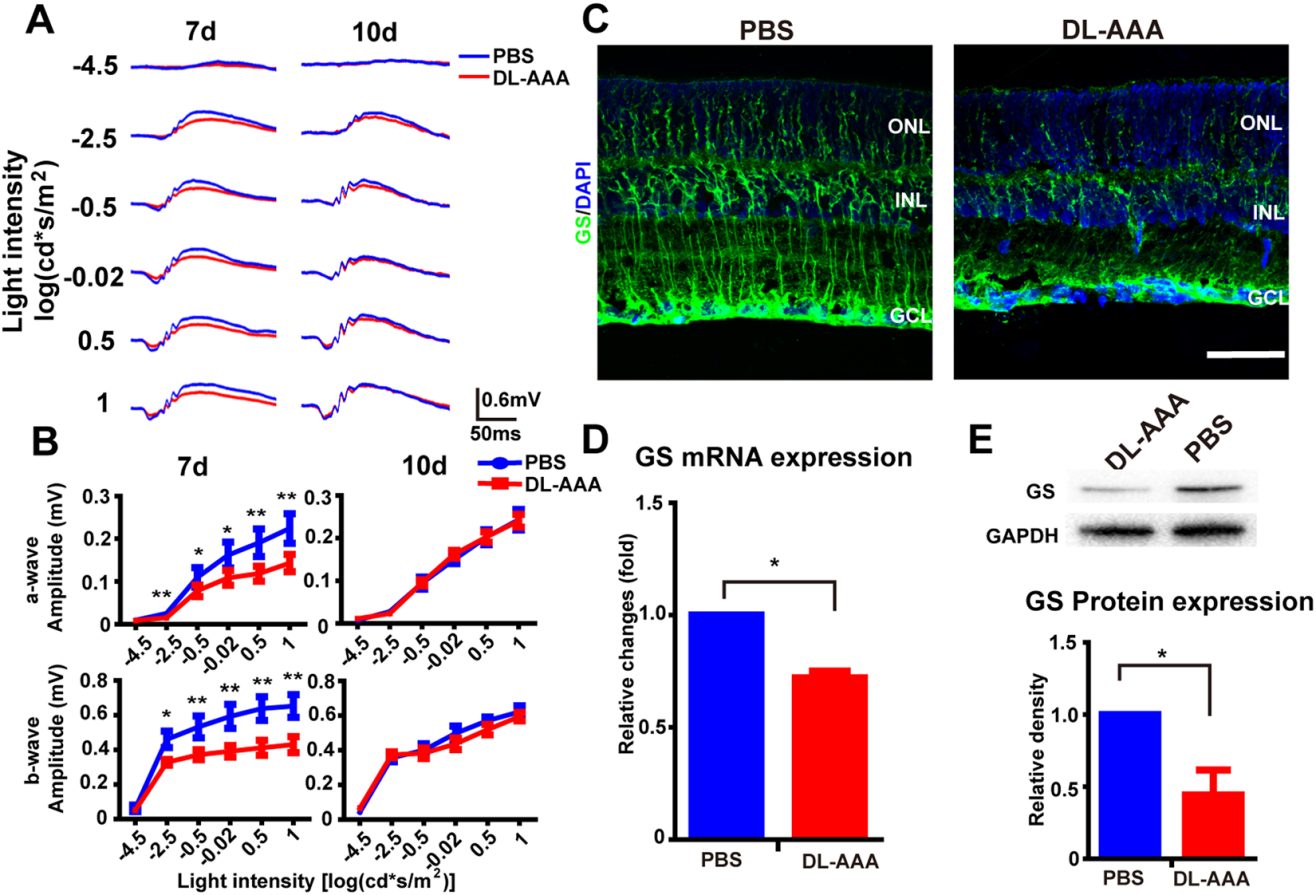
Subretinal DL-AAA injection caused reduced ERG a-/b-wave amplitude and reduced expression of GS. (**A**) Representative light-evoked ERG waveforms measured at 7 days and 10 days following subretinal injection of DL-AAA (*red traces*) or PBS control (*blue traces*) with six different light intensities (from −4.5 to 1 log(cd*m/s^2^)). (**B**) *Top row:* Average stimulus-response curves for a-wave amplitude in DL-AAA treated and PBS-treated eyes at 7 and 10 days post-injection (n = 6 per data point). *Bottom row:* The same for b-wave amplitude. (**C**) Expression of GS protein (*green*) 7 days after injection of PBS (*left*) or DL-AAA (*right*). Nuclear counterstain with DAPI (*blue*). Scale bar = 50 µm (**D**) GS mRNA expression in Müller cells following DL-AAA treatment (n = 3 per group). (**E**) *Top:* Representative western blotting for GS protein expression following DL-AAA treatment. GAPDH as loading control. *Bottom*: Quantification of GS protein expression by western blotting (n = 3 per group). Data are shown as mean ± SEM. *p < 0.05, **p < 0.01. GCL, ganglion cell layer; INL, inner nuclear layer; ONL, outer nuclear layer.

To determine whether the function of photoreceptors was affected by GS inhibition, we injected the glial toxin DL-α-aminoadipate (DL-AAA), a GS inhibitor, subretinally into one eye of RDY rats (control of RCS rats) and phosphate-buffered saline (PBS) into the contralateral eye, as a within-animal control. We then recorded ERG from the eyes at 7 and 10 days post-injection (n = 6 for each group). At 7 days post-injection (Fig. 1A and B, *left column*), the amplitude of both the a-wave and b-wave in the DL-AAA group was lower than the PBS group at the five highest light intensities (*p* < 0.05 for both a-wave and b-wave at these intensities, by paired T-test), but the ratio of b-wave to a-wave was unchanged (Fig. S1A). At 10 days post-injection, no significant differences were found between the DL-AAA group and the PBS group.

Because DL-AAA has other effects on the glutamate-glutamine cycle, beyond GS inhibition^28–31^, we used another specific GS inhibitor, L-methionine sulfoximine (MSO), to test the relationship between the disruption of GS and the attenuation of ERG waves (Fig. S2). Our results confirmed that both a- and b-waves decreased 7 days after subretinal injection of MSO, but the ratio of b-wave to a-wave was unchanged (Fig. S1B).

A decrease in expression of GS in Müller cells following DL-AAA injection has previously been reported^32, 33^. To examine this further, cryostat sections of rat retinas, 7 days after injection, were treated with fluorescent-labelled anti-GS antibodies, and the immunostaining pattern was measured using a fluorescence microscope. A decline in expression of GS protein was observed at day 7 post DL-AAA injection (e.g. Fig. 1C). These results were further supported by reverse-transcription polymerase chain reaction (RT-PCR) and western blotting experiments (Fig. 1D and E). In summary, GS expression can be reduced by DL-AAA administration, and thus the glutamate-glutamine cycle may be compromised by DL-AAA administration.

**Figure 2:**
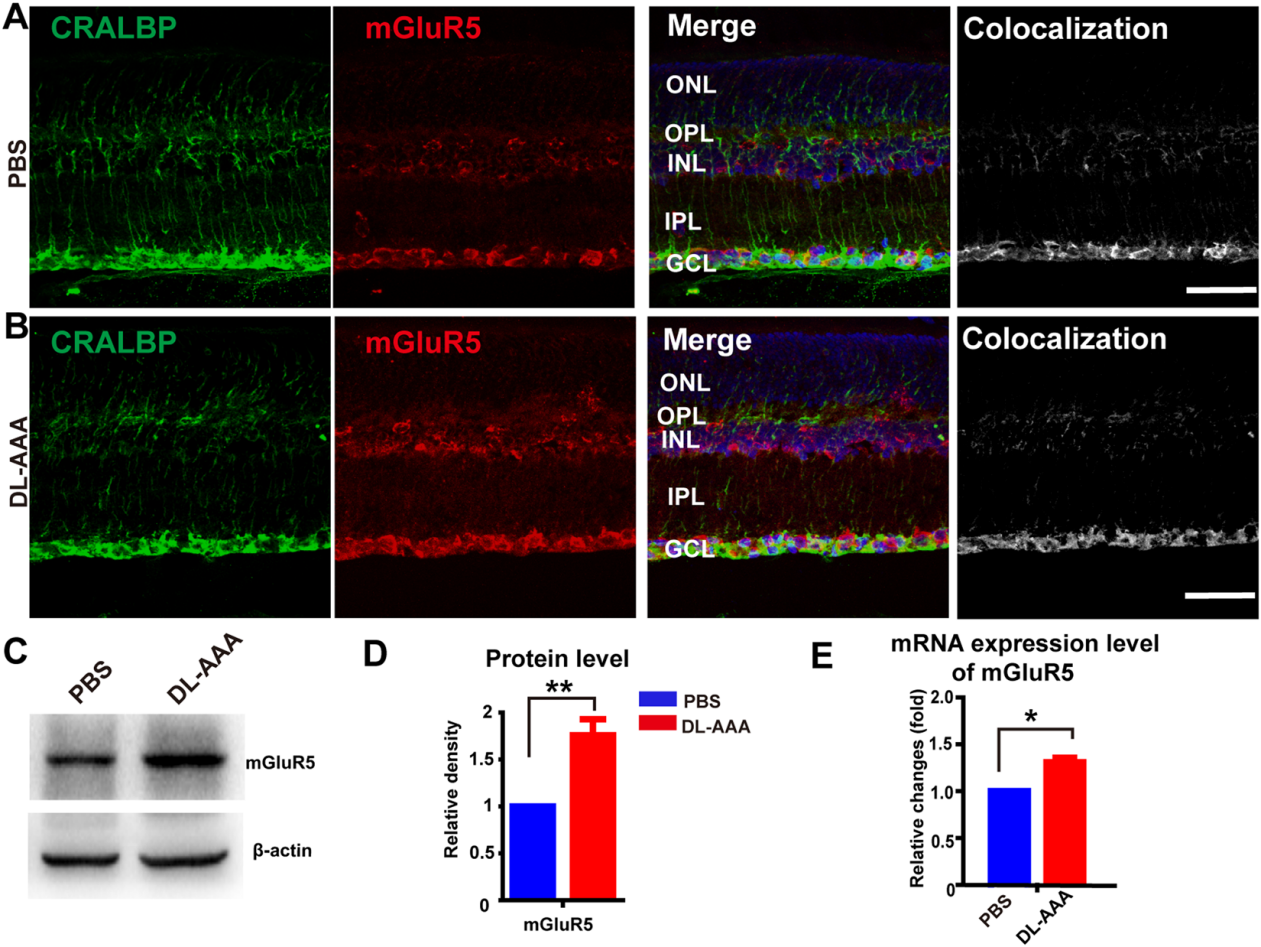
Subretinal DL-AAA injection caused increased expression of mGluR5. (**A**) Representative immunostaining for mGluR5 (*red*) and CRALBP (*green*) in a PBS-treated retina. Colocalization of the two (*right column*) is indicated by brightness of grey pixels. Scale bar = 50 µm. (**B**) Same as **A** but for a DL-AAA treated retina. (**C**) Representative western blotting for mGluR5 expression 7 days following DL-AAA treatment. β-actin as loading control. (**D**) Quantification of mGluR5 protein expression by western blotting, 7 days following DL-AAA treatment (n = 3 per group). (**E**) mGluR5 mRNA expression 7 days after subretinal injection of either DL-AAA (or PBS control). Data are shown as mean ± SEM. *p < 0.05, **p < 0.01. ONL, outer nuclear layer; OPL, outer plexiform layer; INL, inner nuclear layer; IPL, inner plexiform layer; GCL, ganglion cell layer.

What causes the changes in the ERG amplitude after DL-AAA delivery? It has been previously demonstrated that GS activity influences the uptake of glutamate^34^ and the recycling of glutamate is disrupted after inhibition of the GS^20^, which may lead to the over-activation of group I metabotropic glutamate receptors expressed on Müller cells in a model of chronic ocular hypertension (COH)^11^. To investigate the molecular pathways in Müller cells that lead to decreased ERG amplitude, we injected DL-AAA, and studied the effect on mGluR5 and mGluR1 expression using immunofluorescence staining, RT-PCR, and western blotting (Figs 2 and S3).

Using immunofluoresence staining, we found that mGluR5 expression was increased 7 days after DL-AAA injection, compared to PBS control (e.g. Fig. 2A and B, *red channel*). Colocalization of mGluR5 (*red*) and CRALBP, a fluorescent stain for Müller glia (*green*), was most prominent in the outer plexiform layer (OPL), inner nuclear layer (INL),inner plexiform layer (IPL) and ganglion cell layer (GCL) (Fig. 2A and B, *right column*).

Western blotting demonstrated that mGluR5 protein expression was 87% ± 19% higher in the DL-AAA treated eyes than the PBS control eyes (*p* = 0.0015, by paired T-test; Fig. 2C and D). Quantitative real-time PCR (RT-PCR) showed that mGluR5 mRNA expression was 31% ± 5.3% higher in the DL-AAA group (*p* = 0.028 vs. PBS control, by paired T-test; Fig. 2E). In support of these results, treatment with MSO produced a similar (60%) increase in mGluR5 mRNA expression 7 days after injection (*p* = 0.0072 vs. PBS, by paired T-test, Fig. S2C).

By contrast, there was no significant effect of DL-AAA on either mGluR1 mRNA transcription (p = 0.7479 vs. PBS control, by paired T-test; Fig. S3), or mGluR1 protein expression (p = 0.1504 vs. PBS control, by paired T-test; Fig. S3). Taken together, these results indicated that inhibiting GS in Müller glia increased their expression of mGluR5, but not mGluR1.

We speculated that the significant increase in mGluR5 expression may be functionally associated with the diminished amplitude of the ERG. To confirm this, an agonist and an antagonist of mGluR5–(R,S)-2-chloro- 5-hydroxyphenylglycine (CHPG) and 2-methyl-6-(phenylethynyl)-pyridine (MPEP) respectively–were injected into the subretinal space of separate groups of RDY rats.

**Figure 3:**
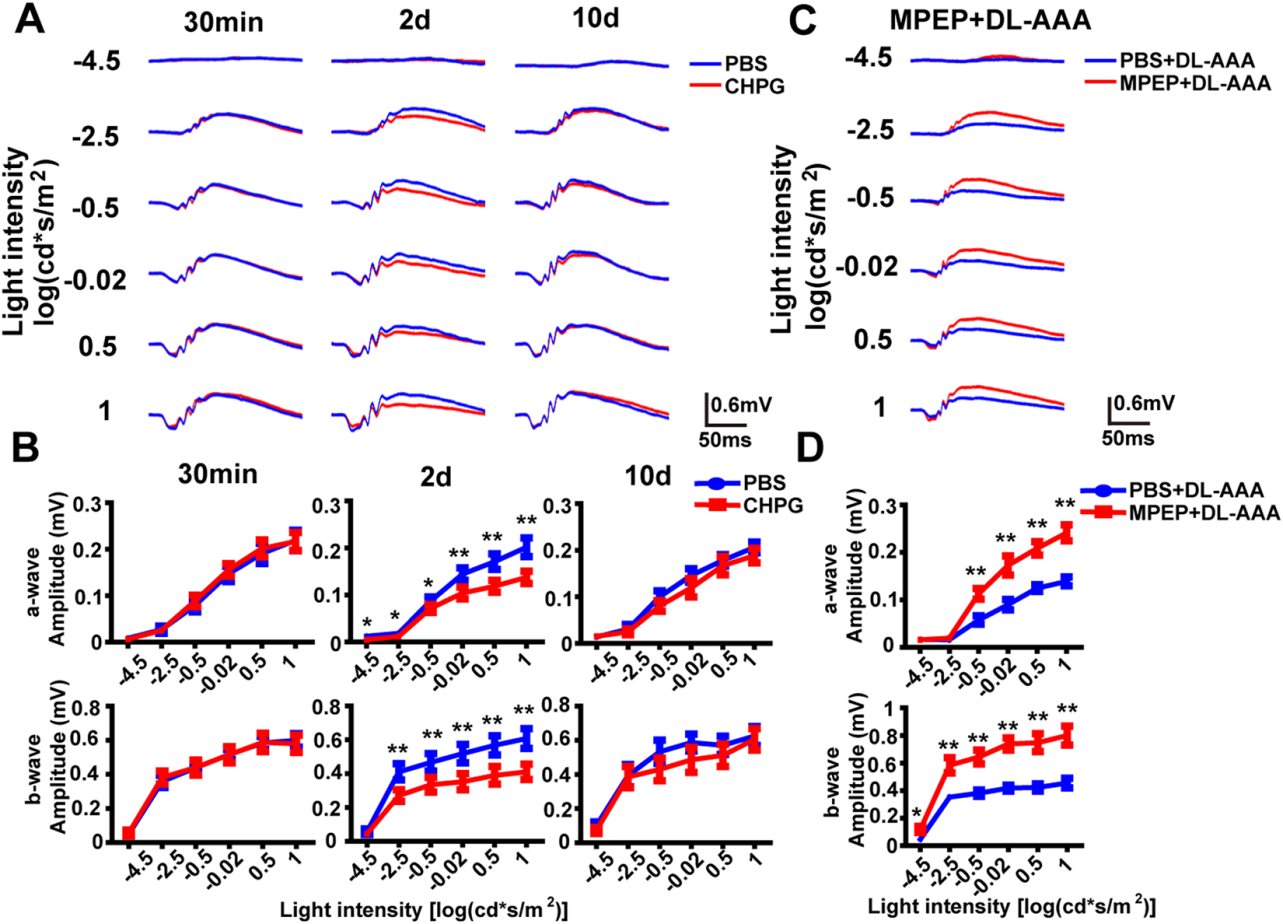
Effects of CHPG and MPEP + DL-AAA on the ERG. **(A**) Example ERG waveforms recorded from CHPG treated eyes (*red*) and PBS-injected eyes (*blue*) at 30 min, 2 days and 10 days post-injection. (**B**) *Top row:* Average stimulus-response curves for a-wave amplitude in CHPG-treated and PBS-treated eyes at 30 min, 2 days and 10 days post-injection (n = 6, 11, 6). *Bottom row:* The same for b-wave amplitude. (**C**) Representative ERG waveforms for the MPEP + DL-AAA group (*red*) and PBS + DL-AAA group (*blue*) at 2 days after MPEP/PBS injection (n = 6). (**D**) *Top:* Average stimulus-response curves for a-wave amplitude in DL-AAA + MPEP and DL-AAA + PBS treated eyes, 2 days after MPEP/PBS injection (n = 6 per data point). *Bottom:* The same for b-wave amplitude. Data are shown as mean ± SEM. *p < 0.05, **p < 0.01.

Firstly, we injected CHPG (or PBS control) and recorded ERG (e.g. Fig. 3A) at 30 min, 2 days and 10 days post-injection (n = 6, 11, 6 rats for each time point, respectively). At 30 min (0.02 days) and 10 days post-injection, no significant differences were found between the two groups in either the a-wave or b-wave amplitude for any light intensity. At 2 days post-injection, both the a-wave and the b-wave amplitudes of the CHPG group were significantly decreased at all except the lowest light intensity (*p* < 0.05 or 0.01 vs. PBS control, by paired T-tests; Fig. 3B), but the ratio of b-wave to a-wave amplitude was unchanged (Fig. S1D,E).

We also performed experiments where we injected DL-AAA *plus* either MPEP or a PBS control in the contralateral eye, and recorded ERG (e.g. Fig. 3C). At 2 days post-injection, both the a-wave and b-wave were larger in the MPEP-treated group (especially at the higher light intensities) than those in the control groups (*p* < 0.01 vs. PBS control by paired T-tests; Fig. 3D). However, the b-wave to a-wave ratio was unchanged (Fig. S1C). We hypothesized that this was because the increased b-wave amplitude was secondary to the increase in a-wave amplitude. To explore this hypothesis, we plotted a-wave vs. b-wave amplitude for data from 93 rats treated with PBS (Fig. S1E). The data were fitted using least squares linear regression, with an R^2^ value of 0.954.

Next, we analyzed the oscillatory potentials (OPs) of the ERG, which are generally thought to originate in the inner retina^35, 36^. Following either DL-AAA, CHPG or DL-AAA + MPEP treatment, no significant differences were seen in the OPs to b-wave amplitude ratio, compared to PBS control (p > 0.05 for all comparisons; Fig. S4).

**Figure 4:**
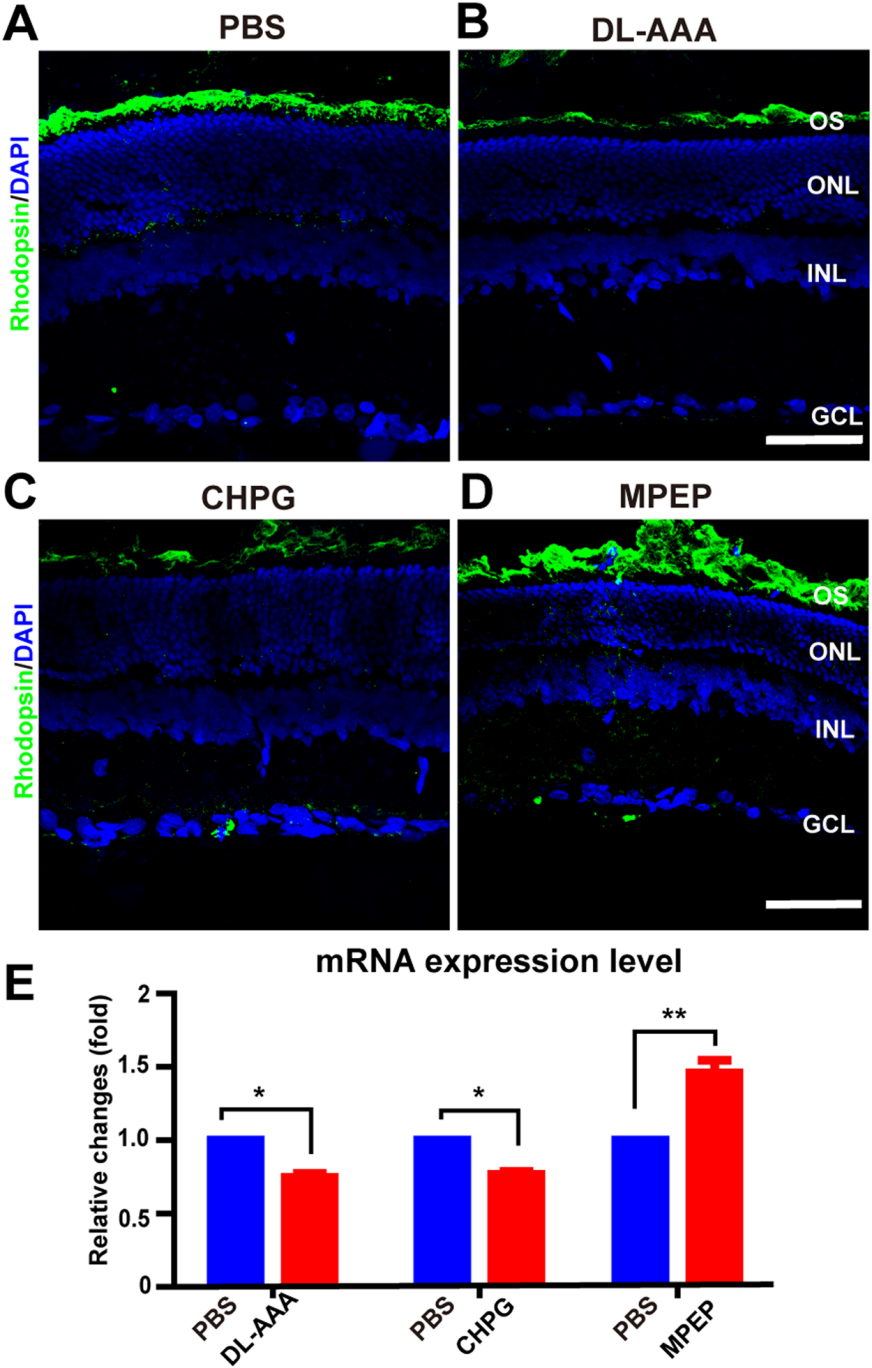
Rhodopsin expression is reduced by DL-AAA and CHPG but increased by MPEP treatment. (**A**) Representative immunofluorescence images showing expression of rhodopsin (*green*) following injection of PBS (control). Nuclear counterstain with DAPI (*blue*). Scale bar = 50 µm. (**B**) Following DL-AAA injection (7 days). (**C**) Following CHPG injection (2 days). (**D**) Following MPEP injection (2 days). (**E**) Rhodopsin mRNA expression following treatment with DL-AAA, MPEP or CHPG, vs PBS control (n = 3 per bar). Data are shown as mean ± SEM. *p < 0.05, **p < 0.01. GCL, ganglion cell layer; INL, inner nuclear layer; ONL, outer nuclear layer; OS, outer segment.

We hypothesized that the changes in the ERG amplitude in response to DL-AAA, MSO, CHPG, or MPEP, were mediated by an alteration in expression of the photopigment protein, rhodopsin. To test this, we examined rhodopsin protein expression using immunofluorescence (Fig. 4A–D), and rhodopsin mRNA expression using RT-PCR, following the drug treatments already outlined above. Rhodopsin mRNA expression was significantly lower in eyes treated with DL-AAA, CHPG or MSO, compared to PBS controls (*p* < 0.05 vs. PBS control, by paired T-test; Figs 4E and S2D). However, rhodopsin mRNA expression was significantly increased following MPEP treatment (*p* = 0.0015 vs. PBS control, by paired T-test; Fig. 4E). Taken together, we concluded that the changes seen in light responses of the outer retina were mediated by changes in rhodopsin expression, and that this was in turn regulated by mGluR5.

**Figure 5:**
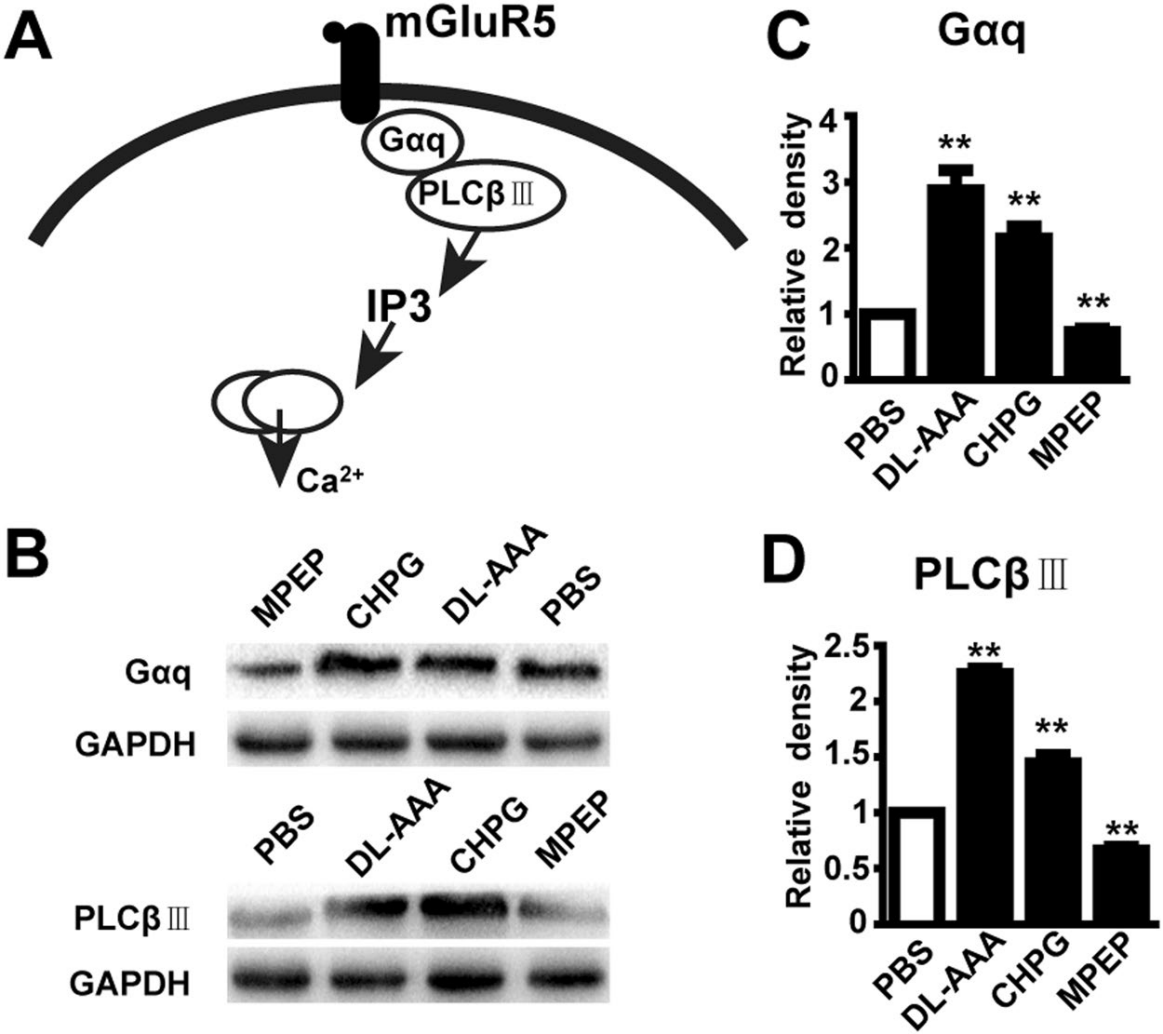
mGluR5 activated the G_αq_ signaling pathway in Müller cells. (**A**) Schematic illustrating the mGluR5 G_αq_ secondary-messenger pathway, following glutamate binding to the receptor. (**B**) *Top*: Representative western blotting showing the expression of G_αq_ following injection with DL-AAA, MPEP or CHPG, vs. PBS control. GADPH as loading control. *Bottom*: The same for expression of PLCβΙΙΙ. (**C**) Quantified G_αq_ expression level following injection with DL-AAA, CHPG or MPEP, vs. PBS control (n = 3 for each bar). (**D**) The same as **C** but for PLCβΙΙΙ expression (n = 3 for each bar). IP3, inositol trisphosphate. **p < 0.01.

Group I mGluRs are found in both neurons and astrocytes, and mediate their effects partly via an intracellular second-messenger cascade that leads to increased concentration of intracellular calcium ions ([Ca^2+^]_i_) (summarized in Fig. 5A). We hypothesized that this second-messenger cascade was being activated in Müller glia by DL-AAA. To test this, we injected RDY rats subretinally with CHPG, MPEP or DL-AAA (and PBS controls in contralateral eyes). Two days (with CHPG and MPEP) or seven days (with DL-AAA) later, we harvested the retinas, and performed western blotting for G_αq_ and PLCβIII on whole-retina lysates. Treatment with CHPG and DL-AAA led to significantly increased expression of both G_αq_ and PLCβIII (*p* < 0.01 for each combination vs. PBS control, by paired T-test, Fig. 5B–D), while treatment with MPEP group led to decreased expression of both proteins (*p* < 0.01 for both vs. PBS control, by paired T-test, Fig. 5B–D). Taken together, these results confirm that pharmacological modulation of mGluR5 altered the expression level of downstream signaling proteins.

### An AQP4/K_ir_4.1/mGluR5 macromolecular complex is expressed on Müller cells

**Figure 6:**
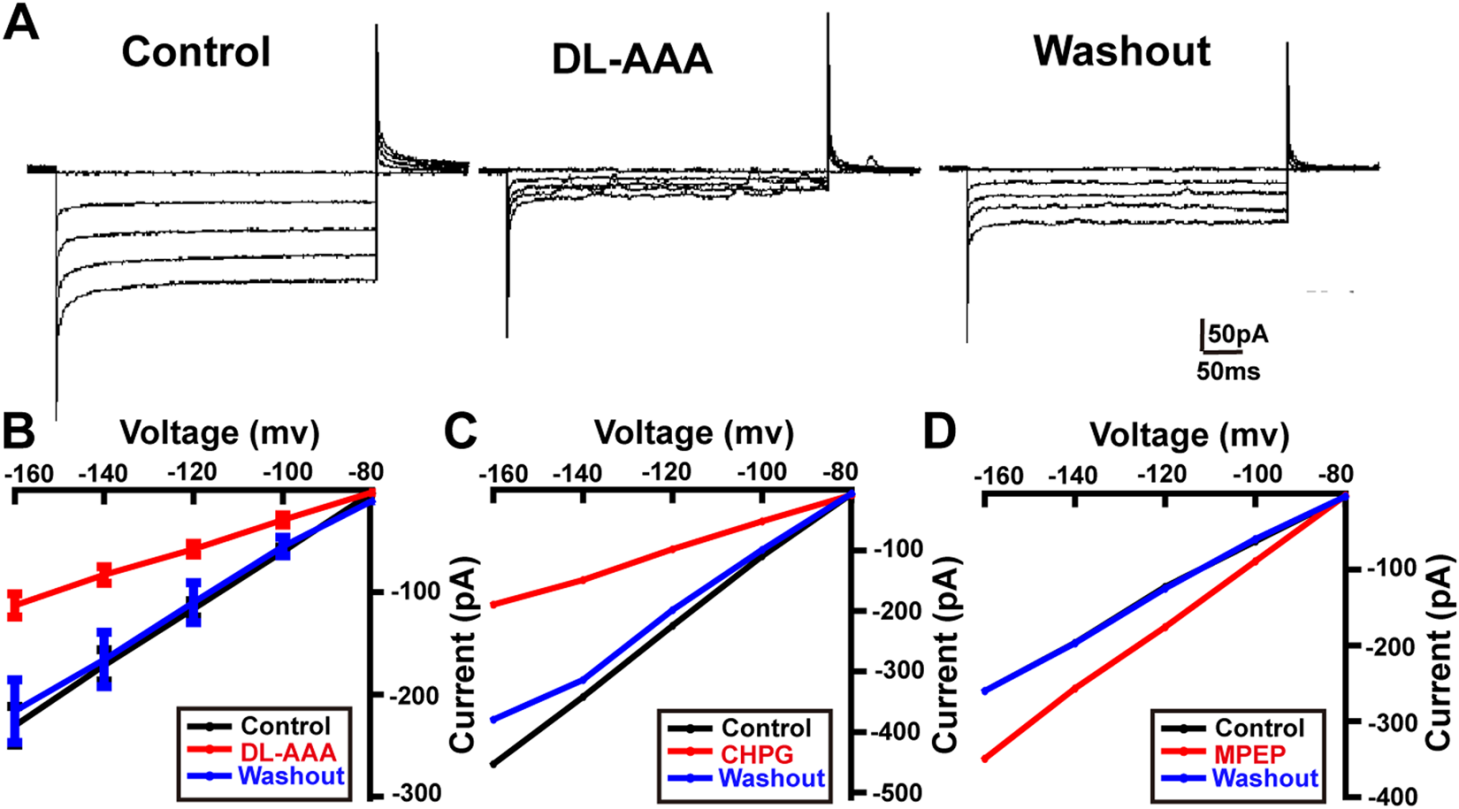
The effects of DL-AAA, CHPG and MPEP on K_ir_ currents of Müller cells. (**A**) Representative whole-cell current traces (under voltage clamp) of cultured Müller cells treated with DL-AAA (in the extracellular medium) at voltage steps of −160 mV, −140 mV, −120 mV, −100 mV and −80 mV. (**B**) Mean I–V relationships of cultured Müller cells treated with DL-AAA in the extracellular medium(*red*), and after washout (*blue*). (**C**) The same as (**B**) but for CHPG. (**D**) The same as **B** but for MPEP. Line charts show means ± SEM.

We next studied the relationship between mGluR5 and inward-rectifying potassium channels (K_ir_) as describe previously^11^. Firstly, we first studied the K_ir_ channel currents using whole-cell voltage-clamp recordings of cultured rat Müller cells, both before and after delivery of DL-AAA (Fig. 6A), MPEP or CHPG (in the extracellular bath medium). Current rose with voltage, but decreased dramatically upon addition of DL-AAA or CHPG. After washout, the amplitudes of the K^+^ currents in the Müller cells were almost fully restored (n = 3, Fig. 6B and C)). In contrast, K^+^ current amplitude was increased after addition of MPEP, and this returned fully to normal after MPEP was washed out (Fig. 6D).

**Figure 7:**
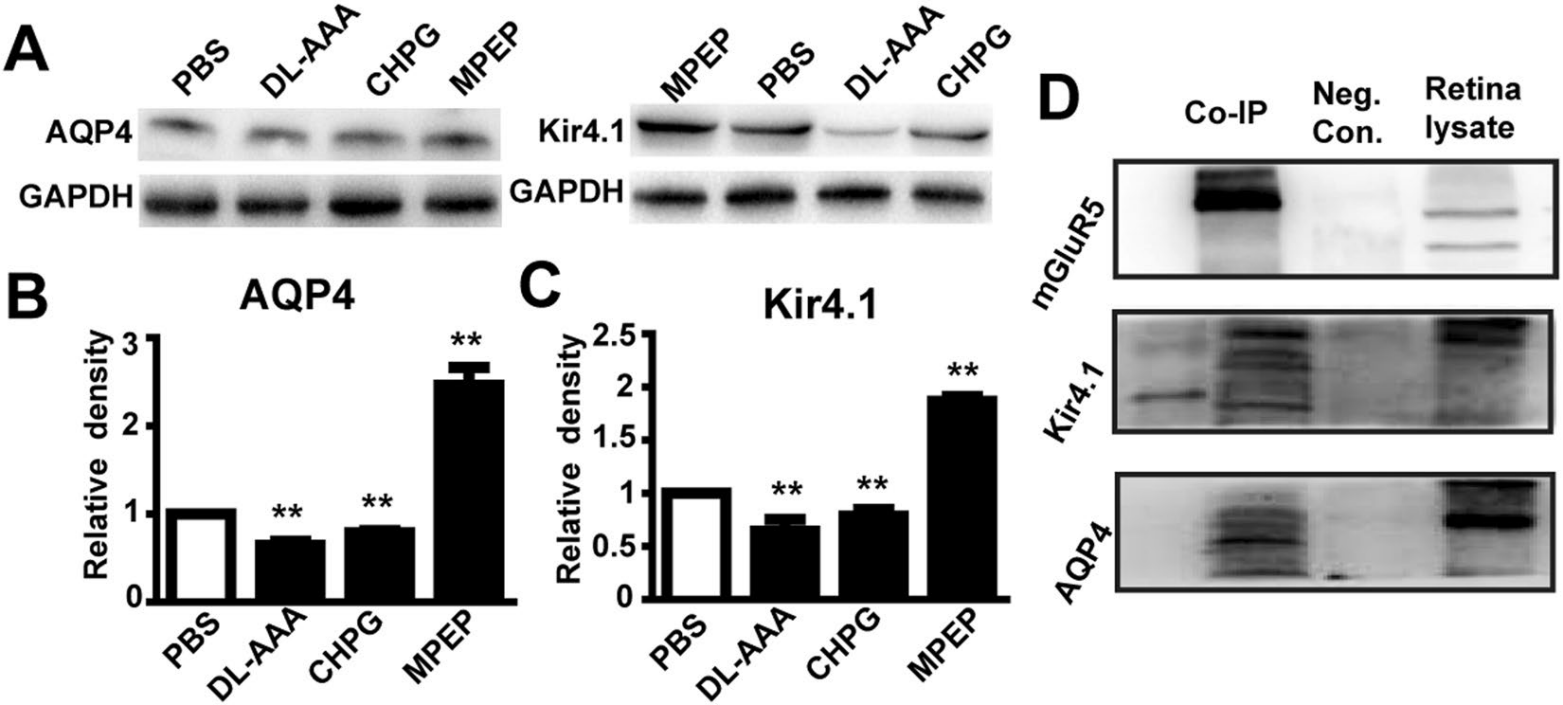
A macromolecular complex is formed by mGluR5, AQP4 and K_ir_4.1. (**A**) Representative western blotting showing the expression of K_ir_4.1 and AQP4 by retinas following the administration of DL-AAA, CHPG or MPEP, vs. PBS controls. GADPH is a loading control. (**B**) Quantification of AQP4 protein expression under the above conditions (n = 3 per bar). (**C**) The same as (**B**), but for K_ir_4.1 expression (n = 3 per bar). (**D**) Representative immunoprecipitation assay for mGluR5, with subsequent immunoblotting for K_ir_4.1 and AQP4. *Neg. Con*., IgG negative control. Retinal lysate was used as a positive control. Bar charts show means ± SEM. **p < 0.01.

We next studied the expression of K_ir_4.1 specifically, as well as aquaporin-4 (AQP4) which is suggested to have a close relationship with K_ir_4.1 on Müller cells^37^. Using western blotting, we found that the expression of AQP4 and K_ir_4.1 was significantly decreased following subretinal injection of DL-AAA or CHPG (*p* < 0.01 for each vs. PBS control, by paired T-test; Fig. 7A–C). By contrast, the expression of AQP4 and K_ir_4.1 was significantly increased following MPEP treatment (*p* < 0.01 for each vs. PBS control by paired T-test; Fig. 7A–C).

Previous studies have shown that, in the adult retina of mouse, AQP4 and K_ir_4.1 proteins are tightly co-localized on the membranes of Müller cells^37, 38^. Furthermore, mGluR5 is known to interact with many molecules, including AQP4, and perform a wide array of vital tasks by forming a macromolecular complex/transporting microdomain in astrocytes^39^. Within the retina, Müller cells are thought to have several functions similar to astrocytes. Therefore, we hypothesized that mGluR5 might also form a complex with AQP4/K_ir_4.1 in retinal Müller cells. To study this, we performed immunoprecipitation assays using an mGluR5 antibody on retinal tissue. These were then immunoblotted with antibodies for AQP4 and K_ir_4.1. We found that AQP4 and K_ir_4.1 co-immunoprecipitated with mGluR5 (Fig. 7D), suggesting that mGluR5 does directly interact with AQP4/K_ir_4.1 molecules in Müller cells.

### Inhibition of mGluR5 restored the ERG b-wave and reduced the expression of GFAP in RCS rats

Our results suggested that Müller cells indirectly regulate the response of photoreceptor cells to light stimulation via modulation of mGluR5. Given the potential therapeutic implications of these findings, we next wanted to study the properties of mGluR5 in a model of RP, a condition of degenerative visual loss. To do this, we studied the Royal College of Surgeons (RCS) rat model, a classical animal model of RP, involving Mer tyrosine kinase (MerTk) gene expression defects^40^.

**Figure 8:**
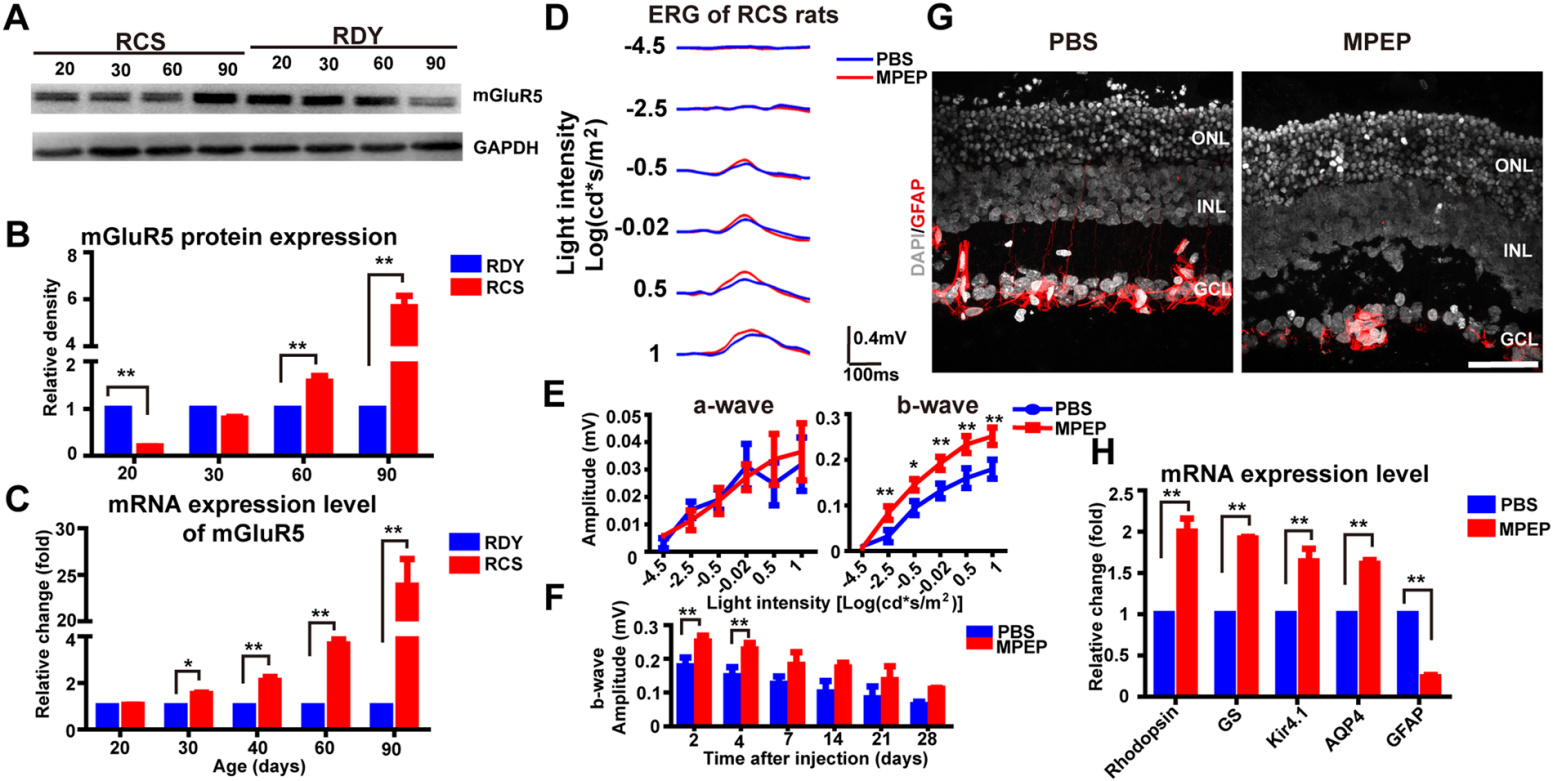
Inhibition of mGluR5 by subretinal injection of MPEP restored the ERG b-wave amplitude and decreased the expression of GFAP in RCS rats. (**A**) Representative western blotting of mGluR5 expression at P20, P30, P60 and P90 in RCS (*left*) and RDY (*right*) rats. (**B**) Quantification of mGluR5 protein expression in RCS rats (*red bars*) vs. RDY rats (*blue bars*) at P20, P30, P60 and P90 (n = 3 for each bar). (**C**) Quantified mGluR5 mRNA expression in RCS vs. RDY rats at P20, P30, P60 and P90 (n = 3 for each bar). (**D**) Example ERG waveforms recorded from MPEP-injected eyes (*red traces*) vs. PBS (control)-injected eyes (*blue*) of RCS rats (P35) at 4 days post-injection. (**E**) *Left:* Average stimulus-response curves for ERG a-wave amplitude in the MPEP-group (*red*) vs. PBS-group (*blue*) at increasing light intensities (n = 7 for each data point). *Right:* The same for b-wave amplitude. (**F**) Amplitude of b-wave (light intensity: 0.5 log(cd*m/s^2^)) following MPEP (*red*) and PBS (*blue*) injection at 2, 4, 7, 14, 21 and 28 days post-injection. (**G**) Example immunolabelling with GFAP (*red*) and DAPI (*gray*) in the retinas of a PBS-injected eye (*left*) and an MPEP-injected eye (*right*) at 4 days post-injection. Scale bar = 50 µm (**H**) Quantified mRNA expression of rhodopsin, GS, K_ir_4.1, AQP4 and GFAP in MPEP-injected eyes (*red*) and PBS-injected eyes (*blue*) at 4 days post-injection (n = 3 per bar). Bar and line graph data show mean ± SEM. *p < 0.05, **p < 0.01. GCL, ganglion cell layer; INL, inner nuclear layer; ONL, outer nuclear layer.

We first tested the expression of mGluR5 in RCS rat retinas at post-natal day twenty (P20), P30, P40, P60 and P90, using western blotting and RT-PCR. We observed significantly increased mGluR5 protein expression in RCS rats at P60 and P90, compared to an RDY control group (*p* < 0.01 at both time points, by unpaired T-test) and significantly decreased expression at P20 (*p* < 0.01 compared to control, by unpaired T-test; Fig. 8A and B). Furthermore, mGluR5 mRNA expression in RCS rats at P30, P40, P60 and P90 was significantly higher than that in RDY rats (*p* < 0.05 or 0.01 at each time point, by unpaired T-test) (Fig. 8C). These results suggested that mGluR5 was overexpressed in RCS rats, particularly at the later stages of retinal degeneration.

Next, we studied the effect of mGluR5 inhibition on the ERG in RCS rats. We studied the eyes of RCS rats that had been subretinally injected with MPEP (with PBS in the contralateral eye as a control) at P35. We performed ERG studies at 2, 4, 7, 14, 21 and 28 days post-injection (n = 7 for each group). At 4 days post-injection, the b-wave amplitudes of the MPEP group were significantly larger than PBS controls at the five highest light intensities (*p* < 0.05 or 0.01, by paired T-tests; Fig. 8D and E). No significant differences were seen in a-wave amplitude. Also, at 7, 14, 21 and 28 days after injection, no significant differences in the b-wave were found between the MPEP-treated eyes and PBS-treated eyes (*p* > 0.05 for each time point; Fig. 8F).

Finally, we studied glial fibrillary acidic protein (GFAP), which is reported to aggravate the degeneration of neurons in the neural degenerated diseases^1^. For example, GFAP expression has previously been reported to be reduced in a rat model of COH after inhibition of mGluR5^11^. We studied whether GFAP expression was reduced in the RCS rat retina by MPEP treatment. We found that there was reduced expression of GFAP at 4 days following MPEP injection (Fig. 8G). Furthermore, GFAP mRNA expression was also significantly reduced following MPEP treatment (p = 0.0005 vs. PBS control, by paired T-test; Fig. 8H). In contrast, the level of mRNA expression for GS, K_ir_4.1, AQP4 and rhodopsin was significantly higher following MPEP treatment (p < 0.01 for each protein vs. PBS control, by paired T-tests; Fig. 8H).

In summary, we found that the amplitude of ERG b-waves were restored on days 2 and 4 after inhibition of the over-activated mGluR5 in RCS rats. And over the same period the expression of GFAP was reduced.

## Discussion

The aim of this study was to determine whether, and how, Müller cells regulate the light response of photoreceptors following the inhibition of glutamine synthetase (GS). Our results demonstrate several findings that have not been previously reported.

The first of these findings is that the ERG a-wave exhibited changes at the various time points following the delivery of DL-AAA or MSO. It has been previously reported that DL-AAA or MSO decrease the b-wave of the ERG, but without an effect on the a-wave^20, 32^. There may be a number of reasons why we have seen a new effect in this study. One reason may be that the toxic effect of DL-AAA is dose-dependent^41^. To ensure that we were effectively inhibiting Müller cells, we examined the effects of various concentrations (7, 70 and 140 μg/μl) of DL-AAA on the ERG (Fig. S5). The amplitudes of the a- and b-waves of ERGs did not change when DL-AAA was administered at the concentration of 7 μg/μl. However, when the concentration reached 140 μg/μl, the amplitudes of the ERG a- and b-waves were significantly decreased, or even disappeared. Additionally, the half-decay time of the normalized waveform (T_1/2d_) –which was determined from the start time to the time at which the normalized b-wave decayed to half of its peak^42^ –was delayed in the DL-AAA injected eyes. These results suggested that not only the outer but also the inner retina was affected by DL-AAA. Therefore, the optimum dose of DL-AAA required for photoreceptor disruption was determined to be 70 μg/μl. Another reason is that the effects of DL-AAA also depended on time, and the functional and morphological changes were differed at the same time point. Our ERG recordings suggested both the a- and b-waves induced by DL-AAA were significantly reduced at 7 days after administration compared with the controls. Finally, these waves began to recover approximately 10 days later. These results are consistent with results reported in carp^32^. However, it has been reported that there is a decrease in GS immunoreactivity at 4 days after DL-AAA injection^33^. Our results demonstrated that the reduction in GS activity could be viewed 1 days after DL-AAA delivery, and they were restored approximately 14 days later (Fig. S6). It was recently demonstrated that the retina fails to recover normal histological morphology following subretinal injection of DL-AAA into mice^41^. A possible explanation is that the retinas between rats and mice differ. Additionally, the different routes of injection and animal models have different influences on the toxic effects of DL-AAA. The most common route of drug delivery to the retina is intravitreal injection. Intravitreally injected DL-AAA causes loss of the ERG b-wave but not the a-wave in the Cyprinus carpio^32^. DL-AAA delivered into the intravitreal space is likely to escape from the vitreous more easily than macromolecular drugs^43^. To overcome the barrier properties of RPE and the retinal inner limiting membrane, subretinal injection can be utilized. Subretinal administration of DL-AAA can alter the blood-retinal barrier in rats and eliminate photoreceptors in monkeys^33, 44^. Our results suggested that ERG a-waves and the expression of GS reduced at 7 days after subretinal injection of 70 μg/μl DL-AAA to the rats. The effects of MSO were the same as that of DL-AAA. It has been previously reported that ERG b-waves decreased 40 min and 90 min after intravitreal injection of MSO, but with slight a-wave enlargement also evident^20^. And the activity of GS was also disrupted at 12 hours after injection^20^.

The second major finding of this study is that mGluR5 contributed to the light responses of the outer retina. In the present study, we demonstrated that mGluR5 was activated after injection of DL-AAA/MSO, which may have been as a result of increased synaptic glutamate, due to reduced glutamate uptake by Müller cells^34^. Selective activation of mGluR5 by CHPG reduced the ERG a-wave amplitude at two days post-injection, which suggests that the functions of photoreceptors were influenced by mGluR5 activity. Additionally, the selective inhibition of mGluR5, by MPEP, rescued the loss of a-waves caused by DL-AAA. These results suggest that mGluR5 may mediate the changes in ERG caused by DL-AAA/MSO.

Our immunofluorescence data showed that mGluR5-postive cells in the retina co-localized with Müller cell markers. In addition, our electrophysiological findings in cultured Müller cells showed that DL-AAA and CHPG decreased the amplitude of K^+^ currents. This is consistent with previous studies which have shown that Group I mGluRs in Müller cells from rat and salamander retina modulate K_ir_ currents, and evoke intracellular calcium waves^11, 45^. These results suggest that mGluR5 is expressed by Müller cells.

Our immunohistochemical data also demonstrated that the expression of mGluR5 was localized in both the OPL and the IPL, which is consistent with a previous report that mGluR5 is present in the dendritic tips of bipolar cells of the OPL and in the amacrine cell processes of the IPL^8^. Therefore, subretinal injection of CHPG may not only activate mGluR5 in Müller cells, but also that expressed elsewhere in the retina. However, the unchanged OP to b-wave ratio and b-wave to a-wave ratio indicate relatively little contribution of neurons in the inner retina to the increased/decreased a-waves induced by MPEP/CHPG. Finally, we found that the expression of rhodopsin was downregulated by the activation of mGluR5, which illustrates a mechanism by which mGluR5 activation can reduce the light response of photoreceptors.

A third major finding from this study is that mGluR5 regulated both K_ir_4.1 and AQP4 in Müller cells, and that these proteins together formed a macromolecular complex. We demonstrated that the expression of mGluR5 was increased after subretinal injection of DL-AAA or CHPG, and the expression levels of its downstream molecules, G_αq_ and PLCβIII, were markedly increased thereafter. These results are similar to the Ca^2+^-dependent PI-PLC/PKC signaling pathway that is involved in the mGluR I-mediated suppression of K_ir_ currents in COH rats^11^. Over-activation of mGluR5 was proven in our experiment to reduce the expression of K_ir_4.1 channels, which is consistent with the statement that the expression of K_ir_4.1 in cultured rat Müller cells is inhibited after activation of the group I metabotropic glutamate receptor^10^.

The decreased expression of K_ir_4.1, caused by mGluR5, may result from the increased expression of G_αq_, because it has been previously reported that the expression of the K_ir_4.1 channel is modulated by multiple neurotransmitters via G_αq_-coupled receptors^46^, and both mutant Ca^2+^-sensing receptors and activated G_αq_ suppress the expression of K_ir_4.1 in HEK-293 cells^47^. It is noteworthy that downregulation of K_ir_4.1 and the opening of cation channels inhibit voltage-dependent glutamate uptake^48^.

Furthermore, we found that the activation of mGluR5 also decreased the expression of AQP4. Previous research suggests that activation of the ERK1/2 pathway decreases the expression of AQP4 in cultured astrocytes^49^, although further studies are needed to explore the interaction between mGluR5 and AQP4. Recent studies have reported that the uptake of glutamate is downregulated in primary cultured astrocytes from AQP4^−/−^ mice^50^. Additionally, the retinas of AQP4^−/−^ mice exhibit lower levels of GS and higher expression levels of glutamate than those of wild type mice under light damage^51, 52^. Therefore, we speculate that decreased expression of K_ir_4.1 and AQP4 caused by the activation of mGluR5 resulted in a decrease in glutamate uptake, and this protected the Müller cells against the toxicity which normally results when the glutamate concentration increases dramatically in the Müller cells after the inhibition of GS^20^.

Fourthly, we demonstrated in our study that a macromolecular complex of mGluR5/Kir4.1/AQP4 exists in Müller cells. Previous studies have consistently shown that mGluR5 interacts with AQP4 by forming a macromolecular complex in astrocytes^39^, and that AQP4 and K_ir_4.1 expression are tightly co-localized in Müller cells^37, 38^. However, the contribution of K_ir_4.1 to the ERG is the slow PIII wave, which is a negative component that is secondary to the a-wave^53, 54^. Delayed b-waves have also been observed in patients who carry the Kir4.1 mutant^55^. Only b-waves are significantly reduced in the AQP4 knockout mice, especially in the older mice^56^. These results of previous studies demonstrate that the individual contributions of K_ir_4.1 and AQP4 to the ERG a-waves can be largely ignored. The decreased light responses of the photoreceptors caused by activating mGluR5 may result from the dysfunction of glutamate uptake and degradation.

Finally, the ERG of RCS rats could be restored by inhibition of the over-activated mGluR5. We observed that mGluR5 was over-activated in the RCS rat during degeneration, especially at late stage, which may result from disrupted glutamate homeostasis^26, 57^. However, the protein level of mGluR5 at P20 and P30 in the RCS rats was reduced when compared with the control groups, which was not consistent with increased mGluR5 mRNA expression level. Further studies are needed to explore the reason for these differences. Most importantly, we found that the b-wave amplitude was increased after inhibiting the upregulated mGluR5 in the RCS rats, which suggests that inhibition of mGluR5 could rescue visual function of RCS rats. The increased b-wave amplitude may result from the improved light responses of photoreceptors. Although no significant differences were found in the a-wave amplitude between the two groups, we speculate that our findings may result from the amplification effects between photoreceptors and bipolar cells^58^. Last, the expression of GFAP, characteristic of gliosis during retinal degeneration, was downregulated after MPEP delivery, which is consistent with a previous report in a COH rat model^11^. This decreased gliosis may also contribute the increased ERG amplitude.

Taken together, we conclude that: 1) the light responses of photoreceptors are reduced after inhibiting GS; 2) mGluR5 and its downstream second messengers are activated after the inhibition of GS; 3) the light responses of photoreceptors and the expression of AQP4 and K_ir_4.1 are likely regulated by mGluR5; and 4) after inhibiting the over-activated mGluR5 in the RCS rats, ERG response amplitudes could be restored and gliosis could be reduced. These results suggest that mGluR5 may be worth considering as a potential therapeutic target in diseases in which there is photoreceptor degeneration.

## Materials and Methods

All procedures were conducted with the approval of the Third Military Medical University Animal Care and Use Committee. RCS-rdy-p^+^ (Royal College of Surgeons rats; postnatal day 20, 30, 35, 40, 60 and 90; either sex) and RCS-rdy^+^-p^+^ rats (RDY rats, postnatal day 20, 30, 40,60 and 90; either sex) were used in this study^40, 59^, and were provided by the Animal Center of the Third Military Medical University, Chongqing, China. Unless specifically mentioned, all rats used in the article were control rats (RDY rats). All experiments were conducted in accordance with the ‘ARRIVE’ guidelines (NC3Rs, London, UK; https://www.nc3rs.org.uk/arrive-guidelines).

### Animal model and drug administration via subretinal injection

All surgical procedures were performed under anesthesia with intramuscular injection of pentobarbital sodium (10 mg/kg), and the fundus was also examined using direct ophthalmoscopy.

DL-α-aminoadipate (DL-AAA) was prepared in accordance with established procedures^32, 33, 44, 60^. Different concentrations of DL-AAA solution (7 μg/μl to 140 μg/μl) were injected into the subretinal space under dim-red light (wavelength >620 nm). Specifically, DL-AAA was injected using a fine glass microelectrode (tip <10 µm) through the sclera at the level of the temporal peripheral retina. In each animal, 4 µl of DL-AAA was injected into the right eye, and the same volume of phosphate buffered saline (PBS) was injected into the contralateral eye as a control.

Similarly, 2-methyl-6-(phenylethynyl)-pyridine (MPEP, 200 μmol), (R,S)-2-chloro-5-hydroxyphenylglycine (CHPG, 200 μmol) and L-methionine sulfoximine (MSO, 20 mM) were individually administered via subretinal injection. Prior to use, CHPG was dissolved in 0.5 M NaOH and then neutralized using 0.5 M HCl. All reagents were purchased from Sigma-Aldrich (St. Louis, MO, USA), except where specifically mentioned.

### Electroretinography (ERG)

ERG was performed as described previously^61^. Briefly, after 12 hours of adaption to darkness, the animals were prepared for recording under dim red-light (wavelength > 620 nm). Light stimuli were delivered at intensities of −4.5, −2.5, −0.5, −0.02, 0.5 and 1 log(cd*m/s^2^). ERG responses were averaged over 3 trials for the two weakest light intensities (and 1 trial used for other stimulus intensities). The inter-stimulus interval was varied between 30 and 120 s, depending on the light intensity.

Data were output to a computer and processed and analyzed using MATLAB 2010b (MathWorks, MA, USA) and Microsoft Excel (Microsoft Corporation, WA, USA). The amplitude of the a-wave was calculated as the maximum negative trough below the baseline, and the b-wave was measured from the a-wave trough to the maximum subsequent positive peak. To extract oscillatory potentials (OPs), raw ERG signals were bandpass filtered at 60–300 Hz (Butterworth filter, 5^th^ order), and the amplitude of each OP was calculated from the amplitude of the largest OP wavelet.

### Immunofluorescence staining

Immunofluorescence staining of frozen sections was performed as described previously^61^. Briefly, the retinas of RCS-rdy^+^-p^+^ (RDY) rats were fixed in 4% paraformaldehyde (PFA)/PBS. Frozen sections, of thickness 10 µm, were cut from retina embedded in optimum cutting temperature (OCT) compound, and preserved at −80 °C. Sections were air dried, washed in PBS for 5 min, and then incubated individually overnight at 4 °C in 0.25% Triton X-100 and 0.25% bovine serum albumin (BSA) in PBS, with the primary antibodies described in Table 1. The sections were then washed three times (5 min per wash) and incubated with the secondary antibody for 1 hour at room temperature (as described in Table 1). All sections were mounted after washing and the nuclei were counterstained with 4’,6-diamidino-2-phenylindole (DAPI).

**Table 1:** Antibodies used in immunofluorescence staining, western blotting and immunoprecipitation.

Antibody	Company	Titre	Species	Cat#	Purpose
Gαq	Santa Cruz	1:50/1:100	rabbit	sc-393	IF/WB
PLCβIII	Santa Cruz	1:50/1:100	rabbit	sc-403	IF/WB
mGluR1	Millipore	1:500/1:1000	rabbit	mab9448	IF/WB
mGluR5	Abcam	1:500/1:1000	rabbit	ab53090	IF/WB
mGluR5	Abcam	1:100/1:200	rabbit	ab27190	IP
AQP4	Abcam	1:100	mouse	ab9512	WB
K_ir_4.1	Santa Cruz	1:100	goat	sc-23637	WB
GFAP	Abcam	1:500	rabbit	Ab7260	IF
GS	Millipore	1:600	mouse	mab302	IF/WB
CRALBP	Abcam	1:200	mouse	ab15051	IF
GAPDH	Cwbio, China	1:2000	rabbit	cw0101	Internal control
β-actin	Cwbio, China	1:1000	mouse	cw0096	Internal control
Secondary antibodies Cy3 (mouse)	Beyotime, China	1:500	goat	A0521	IF
Secondary antibodies Cy3 (rabbit)	Beyotime, China	1:500	goat	A0516	IF
Secondary antibodies Cy3 (goat)	Beyotime, China	1:500	donkey	A0502	IF
Secondary antibodies Cy3 (mouse)	GeneTex	1:500	donkey	GTX85338	IF
Secondary antibodies FITC (goat)	Santa Cruz	1:200	donkey	SC-2024	IF
Secondary antibodies FITC (mouse)	Zhongshan Goldenridge, China	1:200	goat	ZF-0312	IF

### Co-immunoprecipitation

For mGluR5 co-immunoprecipitation, retinas were briefly sonicated in 2 ml radio immunoprecipitation assay (RIPA) buffer (PBS with 1% NP-40, 0.5% sodium deoxycholate and 0.1% SDS) containing 1 mM phenylmethanesulfonyl fluoride (PMSF), a cocktail of protease inhibitors and phosphatase inhibitors (1 mM levamisole, 2 mM Na_2_VO_3_, 1 mM NaF). Anti-mGluR5 antibody (described in Table 1) was incubated with the crude retinal lysate and immunoprecipitated with protein-G agarose and antibodies, according to the manufacturer’s instructions (shown in Table 1). Sodium dodecyl sulfate polyacrylamide gel electrophoresis (SDS-PAGE) and immunoblotting were performed as described below.

### Quantitative real-time PCR and western blotting

To detect mRNA and protein expression levels of GS, rhodopsin, mGluR1, mGluR5, K_ir_4.1, AQP4 and GFAP, we performed RT-PCR and western blotting, as has been described previously^61^. For RT-PCR, the primer pairs used are described in Table 2. The change in expression in the target gene in the experimental group compared to the control group was calculated as a ‘fold’ change, as follows:

$${\rm{fold}}\,{\rm{change}}={2}^{-({\rm{\Delta }}\mathrm{CT},{\rm{Tg}}-{\rm{\Delta }}\mathrm{CT},{\rm{control}})}.$$

For quantitative western blotting, an Odyssey system (V1.2.15; Li-Cor, NE, USA) was used to scan the nitrocellulose membranes for GS, rhodopsin, mGluR1, mGluR5, Kir4.1, AQP4 and GAPDH or β-actin bands. The relative level of each of these proteins was obtained by calculating the ratio of the protein to GAPDH (or β-actin), according to the densitometry for semi-quantification (Full-length blots/gels are presented in Supplementary 2).

**Table 2:** Design of RT-PCR primers.

Genes (rat)	Forward primer	Reverse primer
mGluR5	ATGGAGAAAGTGGGATGGAGGCTT	ACCACCCGGGCTTTAGGTAAATGA
GS	TCACAGGGACAAATGCCGAG	GTTGATGTTGGAGGTTTCGTGG
Rhodopsin	GCAGTGTTCATGTGGGATTG	CTGCCTTCTGAGTGGTAGCC
K_ir_4.1	CAAAGATTGCCCGGCCAAAGAAGA	TGGGTTTGAAGCAGTTTGCCTGTC
AQP4	GGACTCGTCTGGAGAGGTATTA	GAAATCTGAGGCCAGTTCTAGG
GFAP	TCCTGGAACAGCAAAACAAG	CAGCCTCAGGTTGGTTCAT
GAPDH	AGACAGCCGCATCTTCTTGT	TGATGGCAACAATGTCCACT

### Primary Müller cell culture

The eyes from P7–8 RCS-rdy^+^-p^+^ rats were enucleated and incubated for 6–8 h in Dulbecco’s modified Eagle’s medium (DMEM, Invitrogen, MA, USA). The eyecups were transferred to dissociation solution (DMEM containing 1% penicillin/streptomycin, 1% glutamine and 20% fetal bovine serum (FBS)) and incubated at 37 °C in a CO_2_ incubator for 1 h. Eyecups were then washed with DMEM containing 10% FBS and 1% antibiotic-antimycotic mixture, and the retinas were dissected with care to avoid contamination of the retinal pigment epithelium (RPE) and ciliary epithelium. Next, the retinas were mechanically dissociated into small aggregates and cultured in DMEM containing 10% FBS. After 7 days, the floating retinal aggregates and debris were removed, leaving the Müller cells attached to the bottom of the dish. The cells were trypsinized and cultured in DMEM containing 10% FBS for another 5 days, to obtain a further-purified population, and this process was performed twice (in total).

### Whole-cell patch-clamp recordings

Whole-cell patch-clamp recordings were performed as previously described^11, 59^. Briefly, cultured Müller cells were continuously perfused with a solution containin 135 mM NaCl, 3 mM KCl, 2 mM CaCl_2_, 1 mM MgCl_2_, 10 mM 4-(2-hydroxyethyl)-1-piperazineethanesulfonic acid (HEPES), 11 mM glucose, 1 mM sodium pyruvate, 0.5 µM tetrodotoxin (TTX), and 10 mM sucrose, adjusted to pH 7.4 with NaOH.

Patch pipettes were made by pulling borosilicate glass tubes (WPI products, FL, USA). The impedence of the pipette was typically 4–6 MΩ after filling with an internal solution containing 20 mM NaCl, 130 mM potassium gluconate, 1 mM CaCl_2_, 2 mM MgCl_2_, 1 mM ethylene glycol-bis(β-aminoethyl ether)-N,N,N′,N′-tetraacetic acid (EGTA), 10 mM HEPES, 0.1 mM guanosine triphosphate-Na, and 2 mM adenosine triphosphate-Mg, adjusted to pH 7.2 with KOH.

Whole-cell membrane currents were recorded from Müller cells using an Axopatch 200B amplifier in voltage-clamp mode (Axon Instruments, CA, USA) and Clampex computer software (Molecular Devices, CA, USA). Signals were low-pass filtered at 1, 2, or 6 kHz (eight-pole Bessel filter) and digitized at 5, 10, or 30 kHz, respectively, using a 12-bit analog-to-digital converter. All recordings were performed at room temperature (20–25 °C).

### Statistics

All quantitative data are presented as the mean and standard error of the mean (mean ± SEM). Means were compared statistically using paired two-tailed T-tests, except for the experiments comparing RCS to RDY (control) rats, where unpaired two-tailed Student’s T-tests were used. Values of *p* < 0.05 were considered significant.

## Electronic supplementary material

supplementary

supplementary2

raw data
