# Supplementary material for: Improved retinal function in RCS rats after suppressing the over-activation of mGluR5
Source: Sci Rep. 2017 Jun 14;7:3546. doi: 10.1038/s41598-017-03702-z (PMC5471183; doi:10.1038/s41598-017-03702-z)
Supplement: Supplementary file 1 — supplementary [file 41598_2017_3702_MOESM1_ESM.doc]

# Improved retinal function in RCS rats after suppressing the over-activation of mGluR5

Jiaman Dai^1,2^, Yan Fu^2,3^, Yuxiao Zeng^2,3^, Shiying Li^#2,3^, Zheng Qin Yin^#1,2,3^

1. Bioengineering College, Chongqing University, Chongqing, 400040, China.

2. Key Lab of Visual Damage and Regeneration & Restoration of Chongqing, Chongqing, 400038, China

3. Southwest Hospital/Southwest Eye Hospital, Third Military Medical University, Chongqing, 400038, China

**^#^** Correspondence should be addressed to:

Shiying Li

Present address: Southwest Hospital/Southwest Eye Hospital, Third Military Medical University, Chong Qing, 400038, China.

Mobile: +86-13648430819

Fax: +86-23-65460711

E-mail: shiying_li@126.com

Zheng Qin Yin

Present address: Southwest Hospital/Southwest Eye Hospital, Third Military Medical University, Chong Qing, 400038, China.

Mobile: +86-13808336957

Fax: +86-23-65460711

E-mail: qinzyin@aliyun.com

**Running title: Activity of mGluR5 regulates photoreceptors**


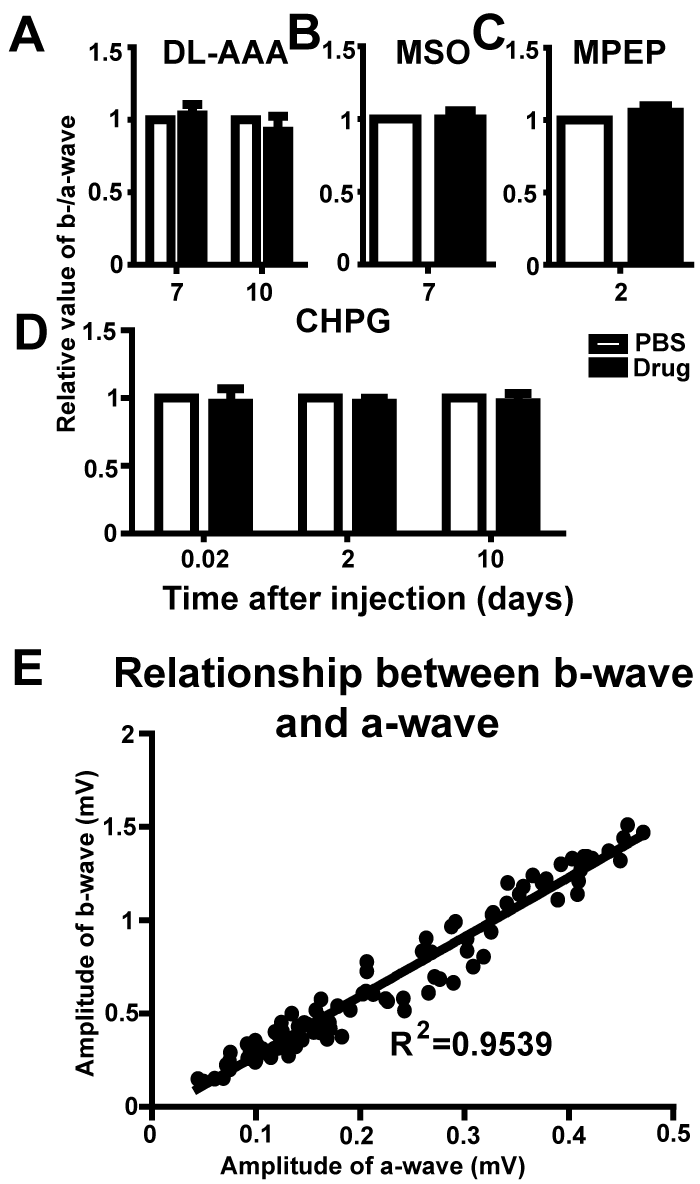


## Figure S1: Linear relationship between a-wave and b-wave amplitude in the ERG of control rats

(**A**) B- to a-wave ratio following treatment with DL-AAA (*black bars*) or PBS control (*white bars*) at 7 and 10 days post-injection (n = 6 per bar). (**B**) Same for MSO at day 7 (n = 6 per bar). (**C**) Same for MPEP at day 2 (n = 6 per bar). (**D**) Same for CHPG at time-points 0.02, 2 and 10 days (n = 6, 11, 6). (**E**) Scatter plot of a-wave vs. b-wave amplitude in rats treated with PBS (n = 93). Regression line and R^2^ value are the result of simple least-squares regression through the points.


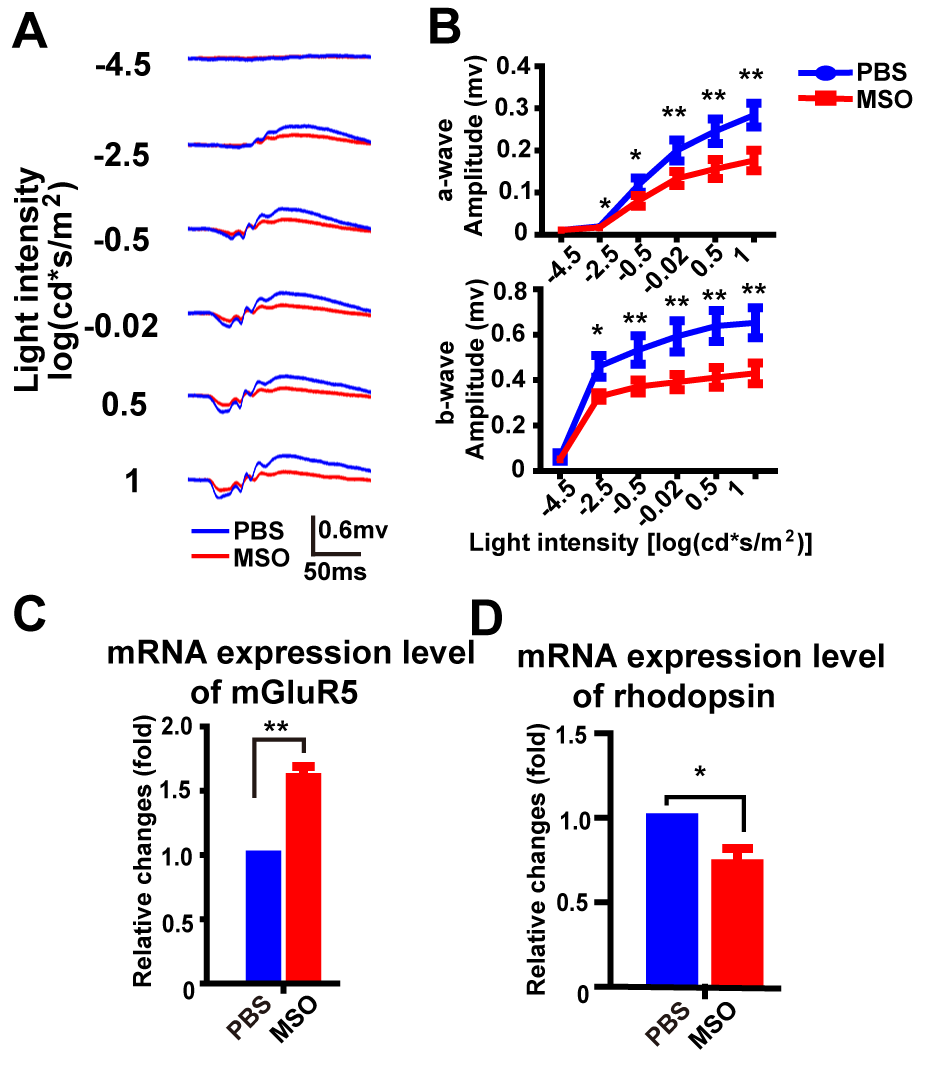


## Figure S2: Changes in ERG features, and in mRNA expression of mGluR5 and rhodopsin, after subretinal injection of MSO.

(**A**) Representative ERG waveforms in response to six different light intensities (−4.5 to 1 log(cd*m/s^2^)), measured from the eyes of rats at 7 days after subretinal injection of MSO (*red*) or PBS (*blue*, control) (**B**) *Top*: Average stimulus-response curves of the amplitude of the a-wave in MSO-treated and PBS-treated eyes at varying light intensity, 7 days after injection (n = 6 per data point). *Bottom*: The same but for b-wave amplitude (n = 6 per data point). (**C**) Quantified mGluR5 mRNA expression level by RT-PCR (n = 3). (**D**) Quantified rhodopsin mRNA expression level by RT-PCR (n = 3). Data are shown as mean ± SEM. **p* < 0.05, ***p*< 0.01.


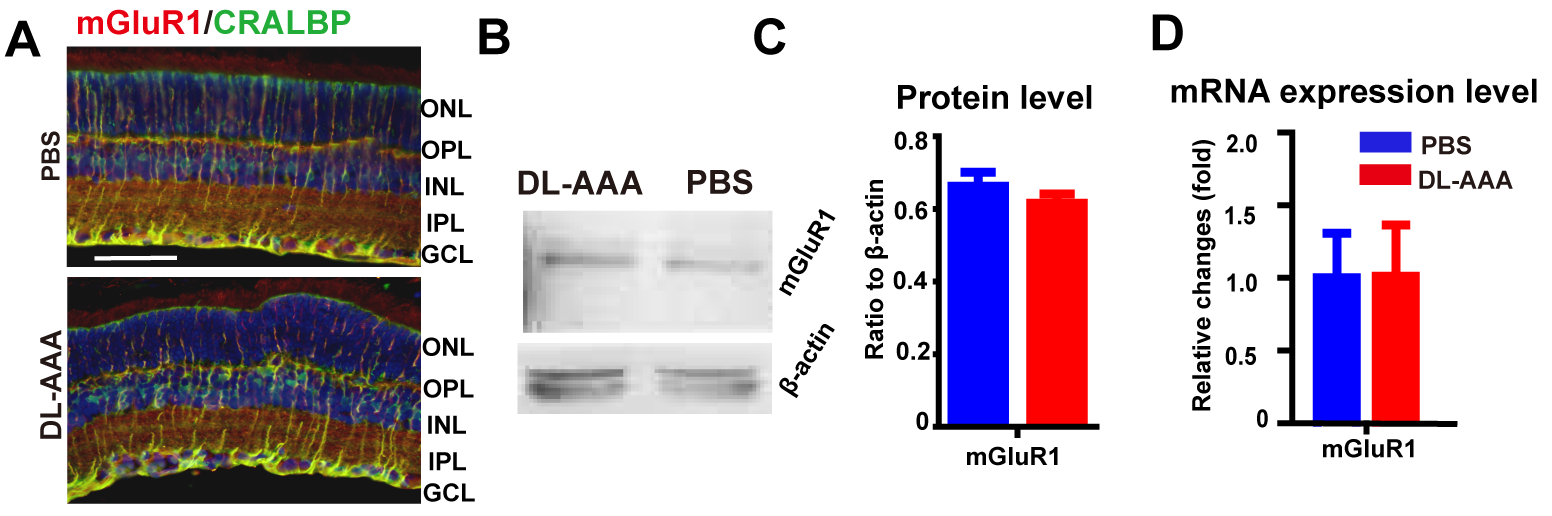


## Figure S3: Expression of mGluR1 in eyes treated with DL-AAA.

(**A**) *Top*: Representative double-labeled immunofluorescence staining for mGluR1 (*red*) and CRALBP (*green*) following injection of PBS control. *Bottom*: The same, but following injection of DL-AAA. Scale bar = 50 µm. (**B**) Representative western blot of mGluR1 protein following injection of DL-AAA or PBS control. β-actin is a loading control. (**C**) Quantification of protein level of mGluR1 following injection of PBS (*blue*) or DL-AAA (*red*) (n = 3 per bar) (**D**) Quantified mGluR1 mRNA expression level by RT-PCR (n = 3 per bar). Data represent mean ± SEM. ONL, outer nuclear layer; OPL, outer plexiform layer; INL, inner nuclear layer; IPL, inner plexiform layer; GCL, ganglion cell layer.


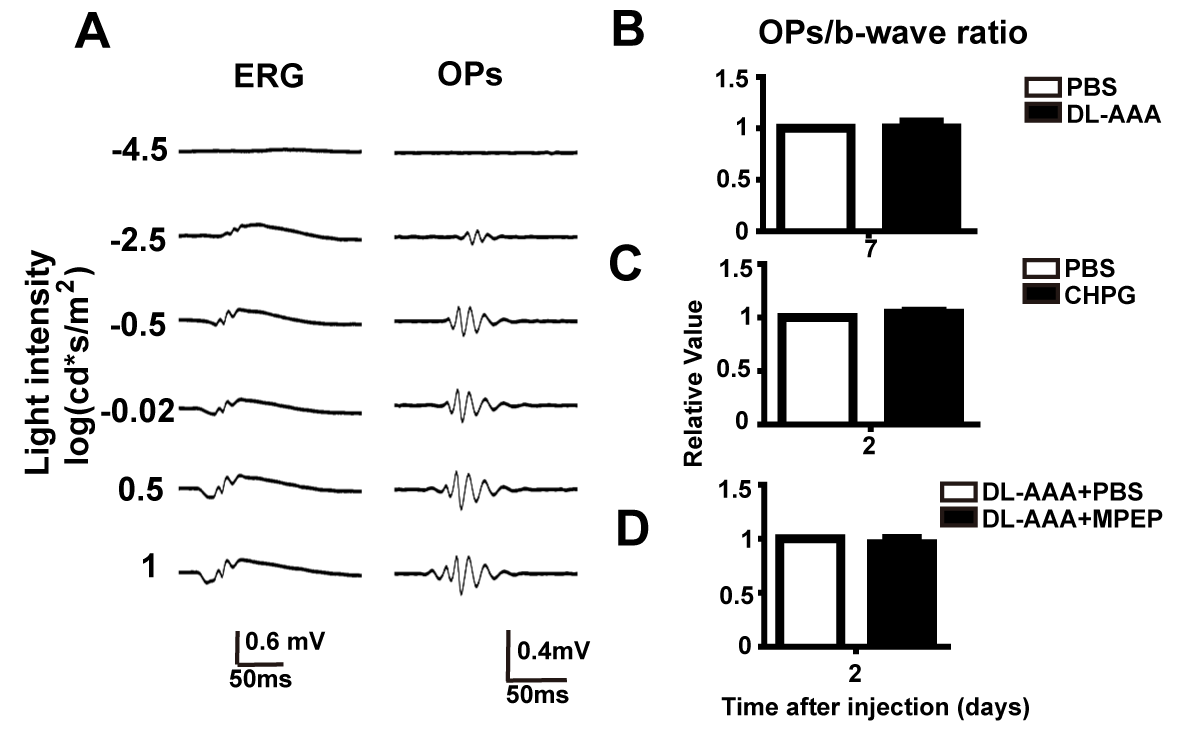


## Figure S4: Effects of DL-AAA, CHPG and MPEP on the OP component of the ERG.

(**A**) *Left column:* Representative ERG waveforms evoked by six different light intensities (from −4.5 to 1 log(cd*m/s^2^)). *Right column*: OP waveforms extracted from the ERG by bandpass filtering at 60–300 Hz. (**B**) OP to b-wave amplitude ratio following treatment with DL-AAA (*black bar*) or PBS (*white bar*) control at day 7 post-treatment (n = 6 per bar). (**C**) Same as **B** but for CHPG at day 2 (n = 11 per bar). (**D**) Same as **B**, but for DL-AAA+MPEP vs. DL-AAA+PBS at day 2 (n = 6 per bar). Data are shown as mean ± SEM.


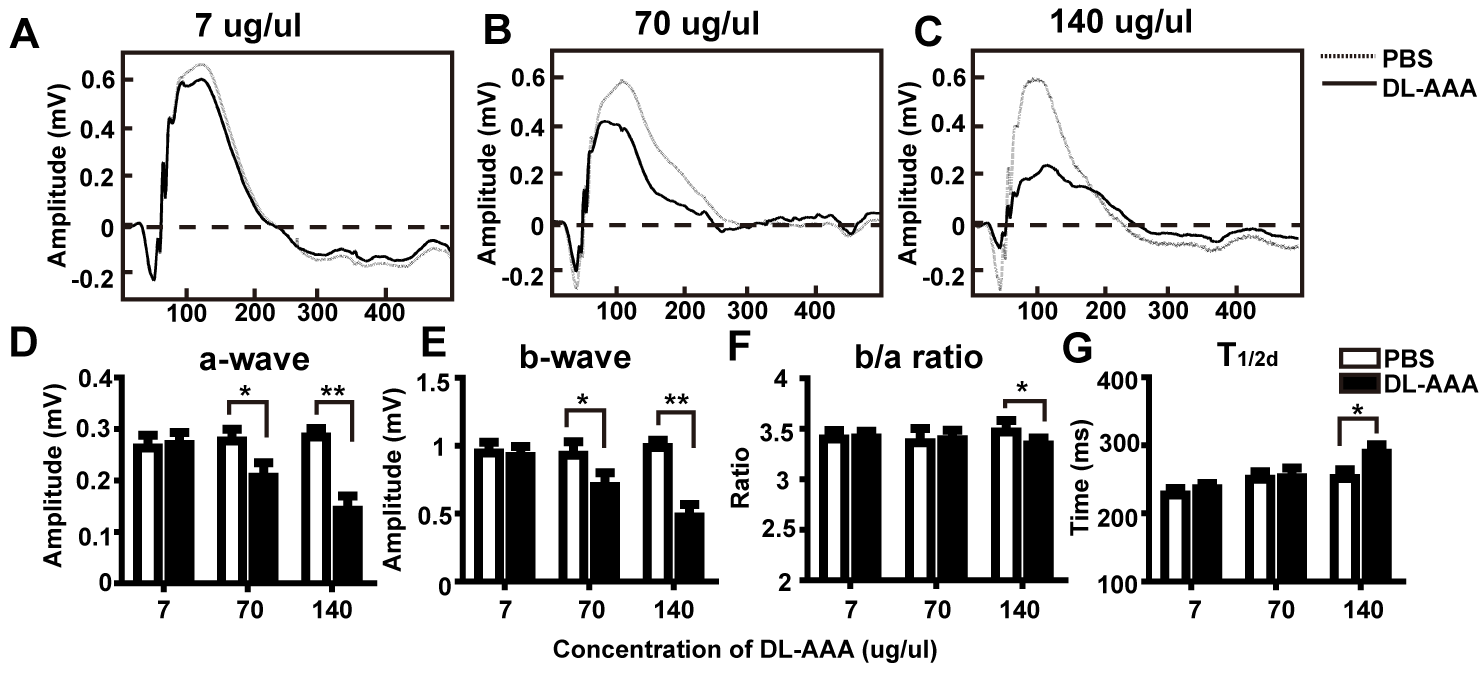


## Figure S5: Different concentrations of DL-AAA produced varying effects in the ERG.

(**A-C**) Representative ERG waveforms (light intensity 0.5 log(cd*m/s^2^)) measured from the eyes of rats treated with three different concentrations of DL-AAA (*solid line*) or PBS (*dashed line*). (**D**) Amplitude of the a-wave with different concentrations of DL-AAA (*black bars*), vs. PBS control (*white bars*) at a light intensity of 0.5 log(cd*m/s^2^). (**E**) The same for b-wave amplitude. (**F**) The same for b/a ratio. (**G**) The same for T_1/2d_ (which is determined from the start time to the time at which the normalized b-wave decayed to half of its peak) (n = 6 for each bar). Data are shown as mean ± SEM. **p* < 0.05, ***p* < 0.01.


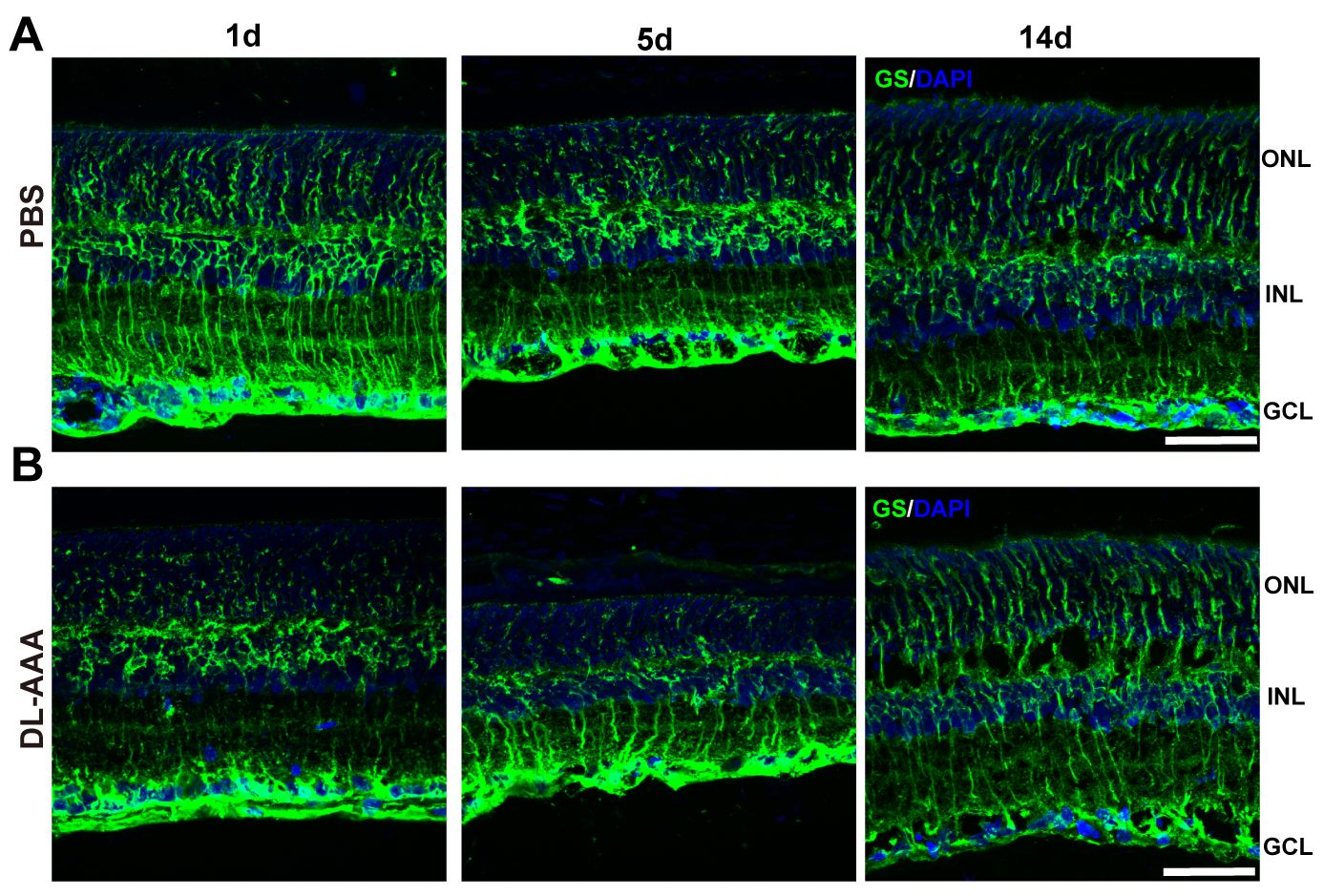


## Figure S6: Expression of GS in retinas at day 1, 5 and 14 after DL-AAA injection.

(**A**) Example of expression of glutamine synthetase (GS, *green*) at day 1 (*left*), 5 (*middle*) and 14 (*right*) post-injection of PBS control. (B) Expression of GS at day 1, 5 and 14 post-injection of DL-AAA. DAPI (*blue*) is a counterstain for cell nuclei. Scale bar = 50 µm. GCL, ganglion cell layer; INL, inner nuclear layer; ONL, outer nuclear layer.
