# Supplementary material for: Improved retinal function in RCS rats after suppressing the over-activation of mGluR5
Source: Sci Rep. 2017 Jun 14;7:3546. doi: 10.1038/s41598-017-03702-z (PMC5471183; doi:10.1038/s41598-017-03702-z)

# Improved retinal function in RCS rats after suppressing the over-activation of mGluR5

Jiaman Dai^1,2^, Yan Fu^2,3^, Yuxiao Zeng^2,3^, Shiying Li^#2,3^, Zheng Qin Yin^#1,2,3^

1. Bioengineering College, Chongqing University, Chongqing, 400040, China.

2. Key Lab of Visual Damage and Regeneration & Restoration of Chongqing, Chongqing, 400038, China

3. Southwest Hospital/Southwest Eye Hospital, Third Military Medical University, Chongqing, 400038, China

**^#^** Correspondence should be addressed to:

Shiying Li

Present address: Southwest Hospital/Southwest Eye Hospital, Third Military Medical University, Chong Qing, 400038, China.

Mobile: +86-13648430819

Fax: +86-23-65460711

E-mail: shiying_li@126.com

Zheng Qin Yin

Present address: Southwest Hospital/Southwest Eye Hospital, Third Military Medical University, Chong Qing, 400038, China.

Mobile: +86-13808336957

Fax: +86-23-65460711

E-mail: qinzyin@aliyun.com

**Running title: Activity of mGluR5 regulates photoreceptors**

Western Blotting

Fig. 1E GS


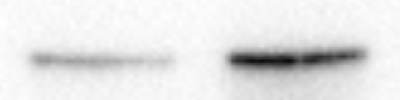


Fig. 1E GAPDH (gs)


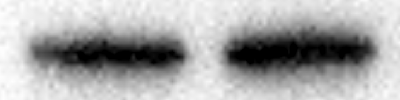


Fig. 2C mGluR5


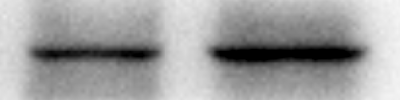


Fig. 2C β-actin (mGluR5)


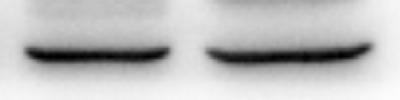


Fig. 5A G_αq_


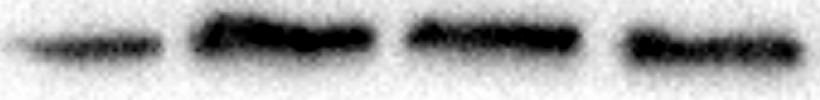


Fig. 5A GAPDH (G_αq_)


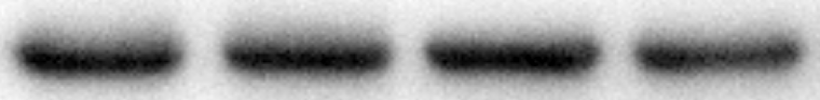


Fig. 5A PLCβIII


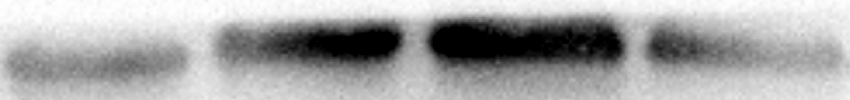


Fig. 5A GAPDH (PLCβIII)


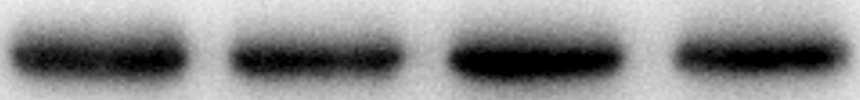


Fig. 7A AQP4


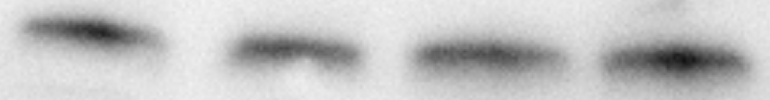


Fig. 7A GAPDH (AQP4)


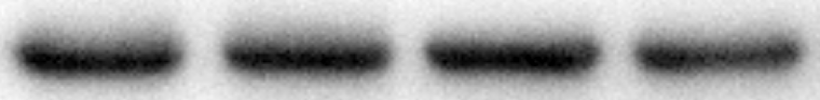


Fig. 7A Kir4.1


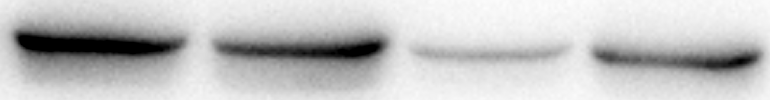


Fig. 7A GAPDH (Kir4.1)


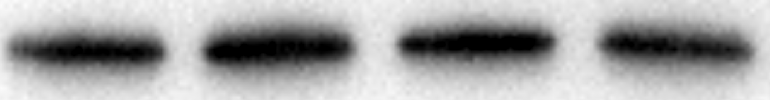


Fig. 8A mGluR5


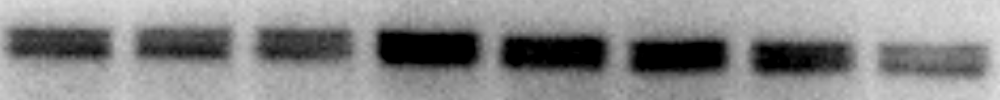


Fig. 8A GAPDH (mGluR5)


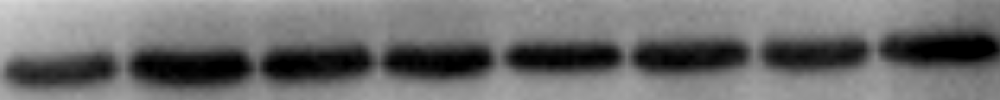

Supplement: Supplementary file 2 — supplementary2 [file 41598_2017_3702_MOESM2_ESM.doc]
